# Peripapillary Pachychoroid Syndrome (PPS): Diagnosing and Treating a Rare Entity

**DOI:** 10.1155/2022/9124630

**Published:** 2022-05-28

**Authors:** Peggy Bouzika, Ilias Georgalas, Maria-Evanthia Sotirianakou, Aristotelis Karamaounas, Chrysanthos Symeonidis, Konstantinos Tyrlis, Stylianos Blounas, Ekaterini Mani, Tryfon Rotsos

**Affiliations:** Retina Service, 1st Department of Ophthalmology, National and Kapodistrian University of Athens, General Hospital of Athens “G. Gennimatas”, Athens, Greece

## Abstract

Two cases with peripapillary pachychoroid syndrome (PPS) along with the challenges concerning correct diagnosis and treatment are presented. In the first case, the patient presented with painless unilateral gradual visual loss. Fundoscopy and optical coherence tomography (OCT) revealed cystoid macular edema (CME) in the left eye (LE), extending from the temporal optic disc margin towards the fovea, with no additional findings. Enhanced-depth imaging- (EDI-) OCT provided additional information and increased choroidal thickness nasally to the macula and pachyvessels in the outer choroidal layer, findings supportive of PPS. Photodynamic therapy (PDT) was applied at the leakage sites. Two months later, CME and subretinal fluid (SRF) had resolved, and VA had significantly improved. In the second case, a patient presented with reduced vision and metamorphopsia bilaterally over the previous 5 days. Fundoscopy revealed CME in both eyes. OCT confirmed the presence of CME in the papillomacular area in the right eye; similarly, CME was recorded in the macula of the LE with SRF located subfoveally. EDI-OCT showed increased choroidal thickness in both eyes. Treatment was administered, originally with dorzolamide eye drops along with eplerenone tablets, and then dexamethasone eye drops that eventually led to significant anatomic and functional improvement. It is important for ophthalmologists to be able to recognize the unique clinical entity of PPS, as its resemblance to disorders with similar features may lead to misdiagnoses and unnecessary, or even incorrect, interventions.

## 1. Introduction

Peripapillary pachychoroid syndrome (PPS) is a relatively novel pathological entity within the pachychoroid disease spectrum (PDS). Although it shares common findings with central serous chorioretinopathy (CSC), it is considered a separate disease. It is characterized by pachychoroid features around the optic nerve head (ONH) and is associated with intraretinal and/or subretinal fluid in the nasal macula. Prominent choroidal pachyvessels, observed on indocyanine green angiography (ICGA), are accompanied by peripapillary retinal pigment epithelium (RPE) mottling and, occasionally, choroidal folds. ONH edema has also been observed in some patients [1].

The aim of this case series is to present 2 cases with PPS along with the challenges concerning correct diagnosis and treatment of this newly recognized disease.

## 2. Case 1

A 72-year-old white male presented with unilateral, painless, and gradual visual loss. Regarding his past ophthalmic history, the patient had been previously treated with intravitreal antivascular endothelial growth factor (anti-VEGF) injections for branch retinal vein occlusion. Specifically, he had received 5 aflibercept injections in his left eye. The patient saw no improvement in his vision, so he opted for another medical opinion.

**Figure 1:**
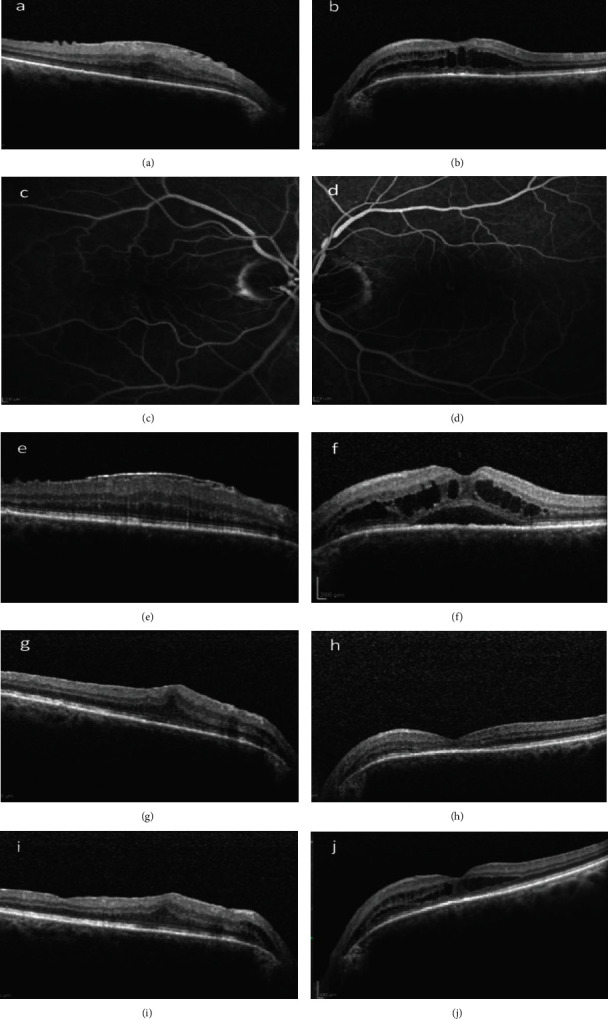
OCT images at presentation showing ERM in the RE (a) and CME in the LE (b). FA of the RE (c) and LE (d) showed very mild hyperfluorescence in the fovea of the LE and mild late staining of the ONH, temporally, in both eyes. Three months later, the OCT of the RE remained unchanged (e), while in the LE there is additional SRF in the fovea (f). OCT images after ERM peeling in RE (g) and topical treatment with dexamethasone and nepafenac drops in the LE (h); the accumulations of SRF and IRF have almost completely resolved. OCTs six months later: the RE (i) is stable, while CME has reappeared in the LE (j).

At baseline, visual acuity (VA) was 20/50 in the right eye (RE) and 20/40 in the left eye (LE). Examination of the anterior segment showed mild cataract in the RE and was unremarkable in the LE. Fundoscopy and optical coherence tomography (OCT) of the macula (Heidelberg Spectralis HRA&OCT; Heidelberg Engineering, Heidelberg, Germany) revealed an epiretinal membrane (ERM) in the RE and cystoid macular edema (CME) in the LE (Figures 1(a) and 1(b)). The CME was localized between the ONH and fovea, extending from the temporal optic disc margin towards the fovea. Apart from a Salus-Gunn sign noted on the superior arcade close to the ONH, no additional findings, such as retinal hemorrhages, exudates, or cotton-wool spots, were observed in the LE. Fluorescein angiography (FA) was also performed; mild hyperfluorescence in the fovea of the LE during the late phase was observed (Figures 1(c) and 1(d)). The patient was placed on a course of dorzolamide eye drops bid in the LE.

Three months later, the CME in the LE still persisted, while subretinal fluid (SRF) had developed in the fovea (Figures 1(e) and 1(f)). A combination of dexamethasone qid and nepafenac 3 mg/ml bid eye drops was initiated in the LE. During the following months, the intraretinal and subretinal fluid started to gradually decrease leading to visual improvement. A small amount of intraretinal fluid (IRF) remained in the papillomacular area of the LE. The patient also underwent pars plana vitrectomy with ERM peeling in his RE with an aim to further improve his vision (Figures 1(g) and 1(h)).

**Figure 2:**
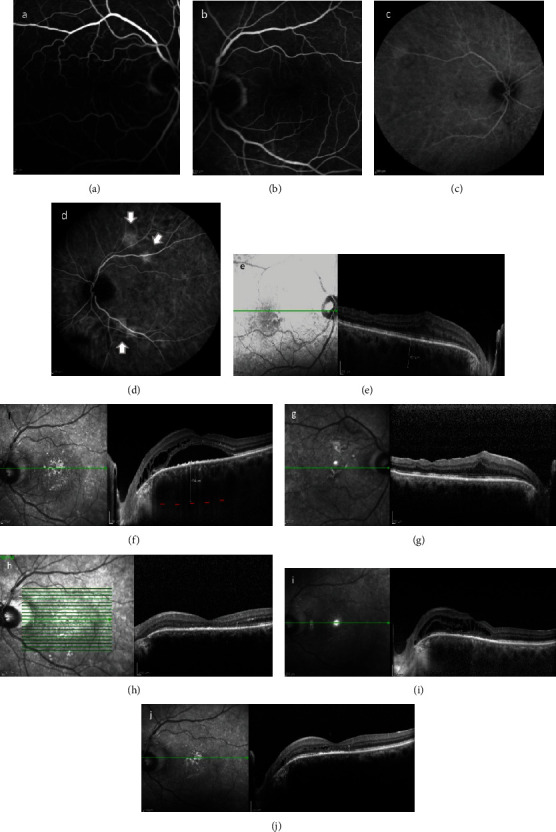
A few months later, there was deterioration in the LE: FA of the RE (a) and LE (b) was noncontributory, while ICGA (c and d) showed hyperfluorescence in 4 foci during the late phase of the exam in the LE corresponding to areas of choroidal hyperpermeability (white arrows, (d)). EDI-OCT revealed increased choroidal thickness [max thickness: 431 *μ*m in the RE (e) and 554 *μ*m in the LE (f)], with pachyvessels (LE > RE) located mostly nasally to the fovea and IRF/SRF accumulation in the fovea of the LE (f). OCT images of RE (g) and LE (h) after PDT treatment in LE, showing resolution of CME and SRF in LE. CME observed in the LE 6 months later (i), and fluid absorption after treatment with dexamethasone drops in the LE (j).

After six months, the CME in the LE increased once again (Figures 1(i) and 1(j)). Systemic administration of acetazolamide tablets (250 mg qid) was added to the treatment. Two months later, SRF redeveloped in the fovea of the LE, leading to a severe VA decrease (counting fingers). Although FA did not reveal significant findings, ICGA showed increased hyperfluorescence due to choroidal hyperpermeability in 4 distinct foci in the posterior pole of the LE, located in the superior and inferior arcades close to the ONH (Figures 2(a)–2(d)). Enhanced-depth imaging- (EDI-) OCT provided additional information, i.e., increased choroidal thickness nasally to the macula and pachyvessels in the outer choroidal layer of the posterior pole, findings supportive of PPS (Figures 2(e) and 2(f)). Photodynamic therapy (PDT) was applied at the leakage sites; intravenous verteporfin (Visudyne, Novartis AG, Basel, Switzerland) was administered over a period of 10 minutes, and treatment was applied 15 minutes after the beginning of the infusion. The light energy used was 25 J/cm^2^ for half-fluence. Two months after the treatment, CME and SRF had resolved (Figures 2(g) and 2(h)) and VA had significantly improved (20/32). Six months later, CME was once more observed in the LE (Figure 2(i)). Dexamethasone eye drops qid were initiated and resulted in almost complete IRF resorption (Figure 2(j)).

## 3. Case 2

A 70-year-old man presented with bilaterally reduced vision and metamorphopsia over the previous 5 days. The patient also suffered from systemic hypertension and coronary artery disease. He was under treatment with methylprednisolone per os, 32 mg per day over the previous 2 weeks, prescribed by his general practitioner for persistent cough.

**Figure 3:**
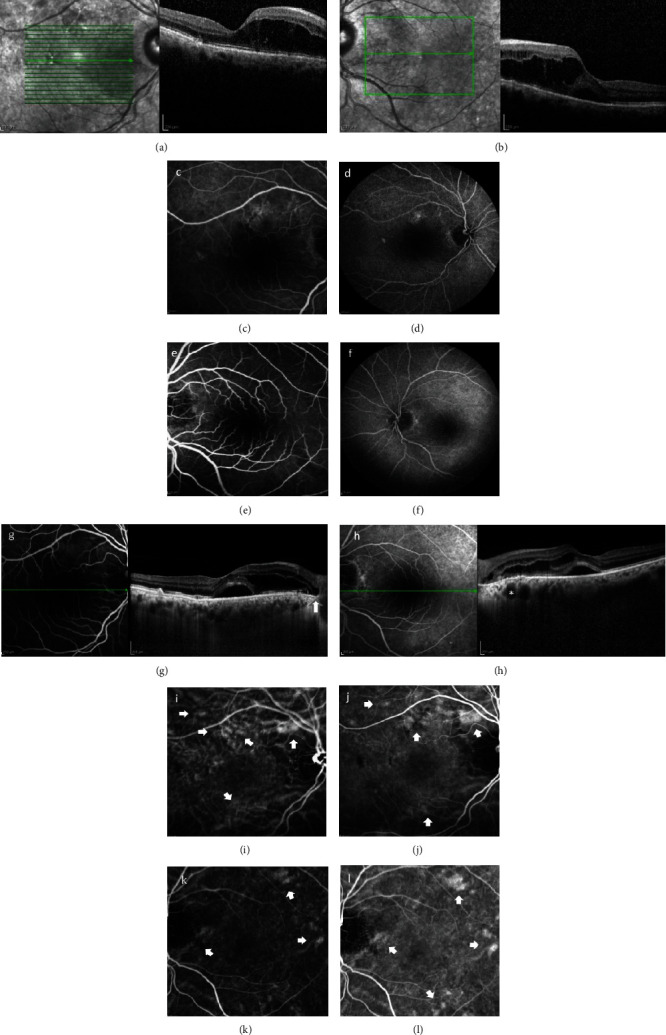
OCT images of RE (a) and LE (b) with CME in the papillomacular area of both eyes and SRF in the LE. FA pictures of RE (c and d) and LE (e and f) showing several small window defects causing hyperfluorescence. Ten days later, the EDI-OCT performed (g and h) showed increased choroidal thickness and pachyvessels (asterisk, (h)) in both eyes (LE > RE) with additional SRF subfoveally in the RE (g); the IRF in the LE was slightly reduced, while SRF persisted (h). PFP was present temporally to the optic disc margin in the RE (white arrow, (g)). ICGA revealed pachyvessels (white arrows), especially superiorly to the ONH in the RE (i and j) and adjacent to the optic disc inferiorly in the LE (k and l), corresponding to hyperfluorescent areas of choroidal hyperpermeability noted during the mid-phase.

VA was 20/63 in the RE and 20/40 in the LE. Ophthalmic examination showed normal intraocular pressure and mild cataract in both eyes. Fundoscopy revealed CME in the posterior pole of both eyes. OCT (Heidelberg Spectralis HRA&OCT; Heidelberg Engineering, Heidelberg, Germany) confirmed the presence of CME in the papillomacular area in the RE; similarly, CME was recorded in the macula of the LE with a small amount of SRF located subfoveally (Figures 3(a) and 3(b)). FA showed 3 small hyperfluorescent areas perifoveally in the RE, and 1 hyperfluorescent area adjacent to the ONH in the LE due to window defects (Figures 3(c)–3(f)). A diagnosis of CSC was hypothesized, and the patient was prescribed dorzolamide eye drops tid and acetazolamide tablets 250 mg bid.

On his follow-up examination 10 days later, there was anatomic deterioration observed in his RE with the appearance of SRF subfoveally; the LE showed a slight improvement, with a small reduction in the amount of IRF present. EDI-OCT showed increased choroidal thickness in both eyes; additionally, there were choroidal pachyvessels adjacent to the temporal edge of the ONH in the LE (Figures 3(g) and 3(h)). ICGA was also performed and demonstrated dilated choroidal vessels, with hyperfluorescent areas in the posterior pole of both eyes, indicative of choroidal hyperpermeability (Figures 3(i)–3(l)). These choroidal alterations led to a new diagnosis of PPS. Topical treatment (dorzolamide drops) remained unaltered with a switch of acetazolamide tablets to eplerenone tablets 25 mg bid.

**Figure 4:**
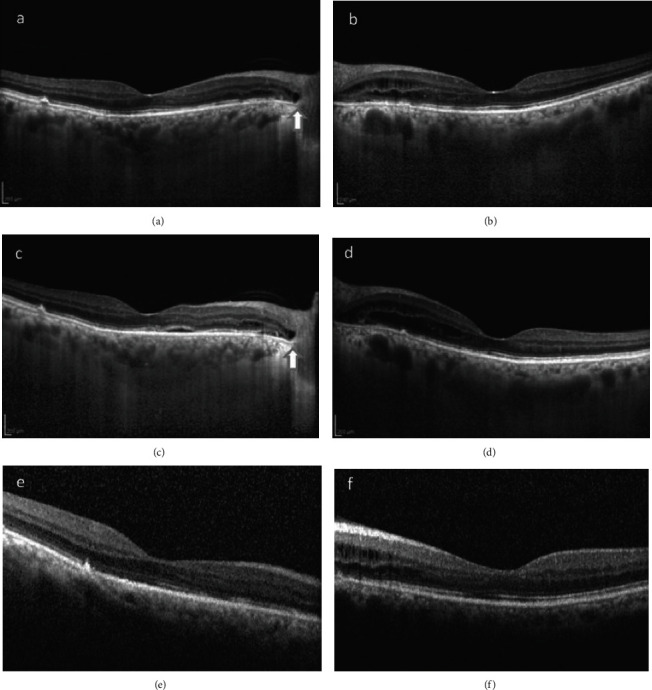
ECI-OCT 3 months after topical and systemic treatment with CAIs and diclofenac drops showed resorption of SRF in both eyes (a and b) with some IRF remaining temporally to the optic disc (LE > RE). A relapse was noted one month later: EDI-OCT showed a small amount of SRF in RE (c) and deterioration with the presence of IRF in the LE (d). OCT after treatment with dexamethasone eye drops: the RE is completely dry (e), and a small amount of IRF remains temporally to the ONH in the LE (f). The arrow denotes the PFP persisting in the RE.

During the following 3 months, VA improved significantly (20/25 bilaterally); the SRF had regressed in both eyes, and only a small amount of IRF remained temporally to the optic disc (Figures 4(a) and 4(b)). One month later, deterioration was observed with a limited quantity of SRF in the RE and an increased quantity of IRF temporally of the optic disc in the LE (Figures 4(c) and 4(d)). The patient was treated with dexamethasone eye drops qid. After a month, OCT scans showed complete fluid absorption in the RE and only a small amount of IRF in the LE (Figures 4(e) and 4(f)). During the following two months, dexamethasone drops were gradually tapered and VA remained stable at 20/25 bilaterally.

## 4. Discussion

This case series highlights the difficulties in diagnosing PPS as well as the variability in both the course of the disease and its response to different treatment modalities. PPS was first described by Phasukkijwatana et al. [2] in 2017 as a distinct pathology within the PDS. Currently, the PDS includes pathologies, such as CSC, polypoidal choroidal vasculopathy, and PPS, conditions which seem to overlap to some extent. The basic features of these choroidopathies are a focal or diffuse increase in choroidal thickness, the presence of choroidal pachyvessels, i.e., dilated choroidal vessels in Haller's layer which lead to attenuation of the inner choroid, and choroidal vascular hyperpermeability. It is important to note that it is not the mere augmentation in choroidal thickness that appears to contribute to the pathogenesis of PDS but rather the increased diameter of the larger choroidal vessels, which may account for the full extent of the choroidal thickness increase [3].

PPS can be differentiated from other disease entities within the PDS due to the presence of pachychoroid features notably in the peripapillary area. Studies in normal eyes have shown that there is a regional variation of choroidal thickness, with the thickest region beneath the fovea and the thinnest nasally [4]. In PPS, the thickest choroidal region is located nasally to the fovea. The IRF and/or SRF that subsequently develop also appear to be extending from the temporal edge of the optic disc towards the macular center. All these findings were present in both cases presented here. In a study by Xu at al [5], data from 35 patients with PPS was used in order to elucidate the peripapillary preference of IRF accumulation in PPS. Specifically, it was noted that, while increased choroidal hydrostatic pressure contributes to diseases, such as CSC, the absence of RPE around the ONH along with the unique peripapillary arteriolar anastomosis present in that area may facilitate the transmission of pressure directly to the inner retina in the peripapillary region, hence facilitating the peripapillary location of PPS.

PPS has also been shown to occur bilaterally and more frequently in hyperopic patients, with crowded optic discs [3]. None of our patients had the latter characteristics, though our second patient did present with PPS findings in both eyes. OCT of the RE revealed a small cystoid space immediately temporal to the optic disc margin, which appears to be a distinct accumulation from the diffuse IRF or SRF present. This “peripapillary fluid pocket” (PFP) has been previously described and is thought to represent a possible entry site of fluid from the choroid to the retina and a biomarker of PPS [5].

Other ocular pathologies causing SRF accumulation, with or without ONH edema, are occasionally considered in the differential diagnosis of PPS; uveal effusion syndrome is strongly associated with nanophthalmic eyes and scleral, rather than choroidal, thickening. Posterior scleritis is a painful entity which causes exudative retinal detachment and choroidal folds, whereas optic disc pit maculopathy may lead to papillomacular detachment, even though the exact source of fluid is still debatable. Our patient had none of the characteristic features of the aforementioned clinical entities.

Currently, there is no established treatment for PPS. Several therapeutic modalities have been tested in the past, such as topical and/or systemic carbonic anhydrase inhibitors (CAIs), intravitreal anti-VEGF injections, and PDT, with variable outcomes [2, 5–7]. In some cases, there was no immediate response to treatment with most patients showing anatomical improvement with VA stabilization. A recent study by Iovino et al. [8] supports the use of PDT for PPS since in their cohort, best-corrected VA significantly improved from baseline to month 3. On the other hand, in a recent PPS case report, it is emphasized that CME in PPS may show significant fluctuations and sometimes observation should be the initial approach especially if vision is not considerably affected [9].

Vision was significantly affected in both our patients, hence rendering observation inadequate as a management option. The least invasive approach was selected with the administration of CAIs topically and systemically; these agents have long been used for the management of CME as they can increase fluid absorption across the RPE [10]. Similarly, eplerenone, a mineralocorticoid receptor antagonist, was used in the second patient, to suppress the effect of increased choroidal thickness and congestion caused by stimulation of such receptors in the choroid [11]. Results were not as favorable as expected, and PDT was employed in order to reduce choroidal hyperperfusion. After PDT, VA in our first patient improved considerably.

The beneficial effect of topical prednisolone on the peripapillary IRF in PPS was recently described by Pothof et al. [12]. In this paper, authors hypothesize that choroidal hyperpermeability resulting in PPS might be secondary to an underlying inflammatory process, hence the positive effect of steroids. Both patients presented here received topical dexamethasone, and both demonstrated a great improvement regarding IRF absorption. This topical treatment may need to be applied over extended periods of time or combined with other therapeutic modalities such as PDT in order to bring about long-lasting results and facilitate the prevention of relapses. In any case, topical steroids, either dexamethasone or prednisolone, may be a simple yet viable treatment for PPS, especially when other options, such as PDT, are unavailable.

## 5. Conclusions

It is important for ophthalmologists to be able to recognize PPS as it constitutes a challenging diagnosis and a unique clinical entity. Its resemblance to disorders with similar features may lead to misdiagnoses and unnecessary, or even incorrect, interventions. Further studies, with a greater number of patients and longer follow-up periods, are warranted for an effective management of PPS.

## Data Availability

Relevant data is available on request. Please contact Dr. Chrysanthos Symeonidis (chrys2209@gmail.com).
